# Data-Driven Patient Engagement to Improve Medication Adherence: A Narrative Review of Targeted Outreach Across Health Systems

**DOI:** 10.7759/cureus.95928

**Published:** 2025-11-02

**Authors:** Chibuzo O Onah, Augustine C Okoye, Taofeek A Yusuff, Edidiong E Abraham, Mwidy Sava M Mounange-Badimi, Tosin Oladosu, Izuchukwu F Okpalanwaka

**Affiliations:** 1 Public Health, Georgia State University, Atlanta, USA; 2 Nursing, Near East University, Nicosia, CYP; 3 Health Administration, Georgia State University, Atlanta, USA; 4 Business Analytics, University of New Haven, West Haven, USA; 5 Pharmacy, Texas Southern University, Houston, USA; 6 Artificial Intelligence and Health Data Science, The University of Texas Health Science Center, Houston, USA; 7 Chemistry and Biochemistry, University of Minnesota Duluth, Duluth, USA; 8 Medical Oncology, Thomas Jefferson University, Philadelphia, USA

**Keywords:** health economics, medication adherence strategies, medication synchronization, risk stratification, targeted outreach

## Abstract

Medication nonadherence undermines chronic disease control and generates avoidable morbidity, mortality, and costs. Between 2010 and 2020, globally, approximately 27% to 40% of patients prescribed antihypertensive therapy were not adherent; poor adherence was associated with 38% higher hospitalization and mortality risks, while similar patterns held for diabetes and lipid‐lowering medicines. This narrative review synthesizes evidence on targeted, data-driven outreach interventions to improve medication adherence. MEDLINE/PubMed, Scopus, and Web of Science were searched for English-language studies (2000-2025, with emphasis on 2019-2025) involving adults or adolescents with measured adherence outcomes (proportion of days covered (PDC), medication possession ratio (MPR), or persistence). Priority was given to randomized trials, economic evaluations, and implementation reports. Interventions varied from low-cost SMS or interactive voice responses to app-based platforms, clinics led by pharmacists, and appointment-based models or medication synchronization (ABM). We identified strategies that reliably improved the PDC or MPR, assessed feasibility across settings, and highlighted equity, privacy, and sustainability considerations. Risk-stratification models demonstrated moderate predictive performance; two-way messaging and pharmacist integration provided consistent benefits that depended on the context, while one-way reminders produced varied effects. Cost‑effectiveness was generally favorable for digital outreach but underreported in low‑ and middle‑income countries. Fairness audits, governance safeguards, and ethical data use remain essential.

## Introduction and background

**Figure 1:**
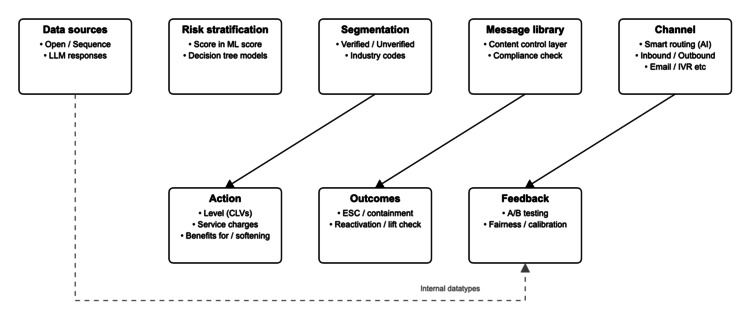
Data to targeting, messaging, channels, actions, outcomes, and feedback Data-to-action pipeline for adherence outreach. Data inputs feed risk scoring and segmentation; message rules drive channeled outreach to trigger actions; and outcomes feed a telemetry loop for A/B calibration and fairness checks. LLM: Large Language Model; ML: Machine Learning; AI: Artificial Intelligence; IVR: Interactive Voice Response; A/B: A/B Testing (split testing for comparing two variants); CLVs: Customer Lifetime Values (patient engagement priority levels); ESC: Escalation (escalating patients to higher intervention levels)

Medication adherence, defined as the extent to which a person’s medication‑taking behavior corresponds with agreed recommendations, remains a global challenge. Large population‑based analyses show that 27%-40% of patients globally prescribed antihypertensive therapy were nonadherent and that poor adherence increased hospitalization and all‑cause mortality by about one‑third [1]. Similar patterns occur in diabetes and dyslipidemia, where poor adherence results in higher hospitalization rates and mortality [2]. Better adherence is also associated with lower risk of stroke and death [2]. Given these consequences, adherence measures, most commonly the proportion of days covered (PDC) and the medication possession ratio (MPR), are incorporated into quality frameworks [3]. A PDC of 80% is widely used as a threshold for adequate adherence to chronic therapy [3]. PDC calculations may overestimate adherence when overlapping refills (“stockpiling”) are allowed and underestimate persistence when switching within drug classes, so methodological choices affect comparability [4]. Claims and electronic health record (EHR) data, dispensing logs, and low‑tech registries enable risk stratification and targeting; recent machine‑learning models predict nonadherence using features such as education level, self‑monitoring, and salt intake [5]. These clinical prediction models use statistical or machine-learning algorithms to estimate individual risk [6]. However, predictive performance is moderate (area under the receiver operating curve (AUROC) ~0.70-0.74), and the models require external validation [5]. Evidence shows that generic one‑way reminders often have modest or null effects [7], whereas segmentation by risk, timing, and two‑way messaging, especially when integrated with pharmacist support, yields more consistent improvements. Meanwhile, algorithmic bias and privacy concerns drive home the importance of fairness audits and governance safeguards [8]. Figure 1 presents an original conceptual framework developed for this review to illustrate the data-to-action pipeline for adherence outreach interventions.

This narrative review aims to: (1) synthesize evidence on data-driven, targeted interventions for improving medication adherence; (2) compare effectiveness across different modalities (short message service (SMS), pharmacist-led programs, medication synchronization); (3) assess feasibility across diverse resource settings; and (4) identify key considerations for equity, privacy, and sustainability in implementation.

## Review

Methods

A narrative review was conducted to synthesize evidence on data-driven, targeted interventions for improving medication adherence. Searches were conducted on MEDLINE/PubMed, Scopus, and the Web of Science for English-language studies involving adults or adolescents. The search terms included "medication adherence," "proportion of days covered," "patient engagement," "SMS," "WhatsApp," "interactive voice response," "pharmacist-led," "medication synchronization," "risk stratification," "machine learning," "claims," "low- and middle-income countries," "equity," and "algorithmic bias." The search emphasized literature from 2019-2025 (≥60% of the included sources) to capture recent developments in digital health technologies, machine learning applications, and implementation evidence. Foundational material (2000-2018) was added for established definitions, theoretical frameworks, and seminal studies. Sources before the year 2000 were included only if they represented landmark contributions to adherence research. Editorials, letters, and abstracts without data were excluded. Titles and abstracts were screened for relevance to targeted outreach with measured adherence outcomes (PDC, MPR, or persistence); reference lists were snowballed. Priority was given to randomized and quasi-experimental studies, economic evaluations, and implementation reports. Study quality was assessed narratively by considering study design, sample size, outcome measurement validity, and completeness of reporting, without formal scoring tools, given the heterogeneity of study designs in this narrative review. Economic papers were appraised for analytic perspective (payer, provider, or societal), time horizon, cost components, and inclusion of sensitivity analyses to assess robustness of findings, following general principles of health economic evaluation reporting. Data were extracted narratively and organized thematically. A meta-analysis was not conducted due to substantial heterogeneity in intervention types (digital vs. pharmacist-led vs. hybrid), outcome definition (PDC vs. MPR vs. persistence), measurement intervals, and study populations across the included literature.

Results

Targeting and Risk Stratification

Effective targeting requires more than a predictive score; it demands validation, monitoring, and guardrails. Standards such as Transparent Reporting of a multivariable prediction model for Individual Prognosis Or Diagnosis (TRIPOD/TRIPOD-AI) emphasize external validation and a clear description of model development and provenance [6]. Most published clinical machine learning models still rely primarily on internal validation; external validation on temporally or geographically distinct cohorts remains uncommon, limiting transportability across systems [9]. Performance can drift over time as formularies, prescribing patterns, and patient behavior change; periodic updating/recalibration and shift monitoring are therefore required [10].

For adherence risk, a minimal viable dataset based on routinely available pharmacy/claims elements, last fill date, days’ supply, gap days, prior adherence, and therapeutic class often provides a strong signal while remaining portable; richer EHR features add cost/complexity with uncertain generalization [11]. Bias checks should extend beyond AUROC to subgroup calibration and error rates (e.g., Positive Predictive Value/False Negative Rate (PPV/FNR) by age, sex, and socioeconomic strata), with remediation when gaps appear [12]. Finally, fairness audits should ask whether operational decisions (e.g., channel eligibility) inadvertently prioritize those with better digital access and document data flows, feature importance, and versioning to sustain clinical trust [13].

Predictive models prioritize outreach by identifying patients at risk of nonadherence. Traditional approaches rely on pharmacy claims (e.g., refill gaps, fill velocity, therapeutic switches) and demographic factors. Machine-learning models incorporating behavioral variables, education, self-monitoring frequency, number of daily supplements, salt intake, and engagement with patient leaflets demonstrate moderate discrimination, with AUC values of around 0.70-0.74. These models can be implemented using non‑clinical data and may serve as scalable tools in primary care, particularly where EHR access is limited [5]. However, they require external validation and calibration to local populations, and there is a risk of algorithmic bias if socio‑economic or racial variables drive predictions [8].

Channels and Interventions

SMS and WhatsApp: Two-way SMS programs allow patients to confirm refills, report obstacles, and receive tailored advice. Meta-analyses indicate modest improvements in adherence, with a relative risk of approximately 1.14, when messages are interactive [14], although heterogeneity persists. In contrast, one-way reminders often show no significant effects; however, platforms like WhatsApp enable richer content and group support, and small trials suggest that they may improve adherence in certain settings, although the data remain limited. High‑resource contexts can integrate app notifications with patient portals, while low‑resource settings rely on SMS or unstructured supplementary service data (USSD).

Evidence varied by condition. In post-acute coronary syndrome, the Text Messages to Improve Medication Adherence and Secondary Prevention After Acute Coronary Syndrome (TEXTMEDS) randomized trial found no improvement in medication adherence with structured texting [15]. By contrast, meta-analyses and trials of two-way SMS that permit replies report modest adherence gains overall and clear benefits in several HIV cohorts (for example, WelTel); systematic reviews and meta-analyses examining SMS interventions that include patients with diabetes also note improvements despite heterogeneity [14,16,17]. Findings in the case of tuberculosis were mixed; a large effectiveness randomized controlled trial (RCT) in Pakistan did not show any benefit of daily two-way SMS reminders on treatment success [18].

Operational constraints frequently blunted effects as shared phones, phone-number churn, literacy and language mismatches, habituation to repetitive messages, and delivery outside decision windows all reduced impact [19]. Designs that asked for a brief reply, captured a specific barrier (e.g., side effects or cost), and triggered a concrete action (auto-refill, call-back, pharmacy switch, or coaching) outperformed broadcast reminders in comparative reviews of one-way vs. two-way messaging [14,17]. To optimize content and timing, pragmatic experiments (A/B or micro-randomized trials) with a pre-specified primary adherence outcome and limited concurrent variants can be used [20,21].

Implementation should match the delivery context. In high-income systems, layering digital prompts with pharmacist-led management and appointment-based medication synchronization yielded more consistent adherence gains than messaging alone [22,23]. In lower-resource settings, two-way SMS/USSD coupled with clinic or community health worker follow-up was feasible and improved visit attendance and adherence in several programs [24,25].

Interactive voice response (IVR) and automated calls: IVR systems provide spoken reminders and allow patients to respond via keypad. Pilot studies demonstrated feasibility and modest adherence gains but highlighted variability in effect sizes. Combining IVR with human follow‑up appeared more effective than automated calls alone. Voice platforms may benefit populations with low literacy, but raise privacy concerns if phones are shared [26].

Pharmacist‑led programs: Pharmacist involvement consistently demonstrated beneficial yet context‑dependent effects. Randomized and quasi-experimental studies showed improvements in PDC and persistence when pharmacists provided medication therapy management, reconciled regimens, and offered coaching [23]. Appointment‑based models or medication synchronization (ABM) synchronized all refills and scheduled pharmacist appointments; they increased adherence and detected drug therapy problems [22]. A 2025 retrospective cohort evaluating electronic outreach with pharmacist support for Medicare Advantage enrollees found that adherence improved for diabetes medications (PDC≥0.8 increased from 83.4% to 87.5%), but not for hypertension or cholesterol drugs [2]. Out of 1,593 electronic messages, 74.4% were opened and 32.4% elicited responses, with patient self‑reported adherence being the most common reply [2]. Patients appreciated outreach but occasionally expressed confusion or irritation [23]. Overall, the program produced mixed results, emphasizing that it required more tailored messaging and timely pharmacist engagement [23].

Pharmacist-led interventions worked through multiple mechanisms [27]. Comprehensive medication reviews identified contraindications, duplications, and dosing errors, enabling deprescribing or simplifying complex regimens [28,29]. Pharmacists counsel patients on technique (e.g., inhaler use), lifestyle modifications, and potential side effects, addressing misconceptions that drive intentional nonadherence [27,30]. In transitions-of-care settings, pharmacists' follow-ups ensured that discharge prescriptions were filled and reconciled with pre-hospitalization regimens [28,29]. ABMs aligned all chronic medications to a single pickup date, reducing cognitive load and transportation costs [22,31]. These services often produced moderate improvements in adherence metrics; however, effects depend on comparator conditions and follow-up length [27]. Trials with usual care comparators and at least six months of follow-up generally demonstrated positive effects, whereas studies using active comparators (e.g., nurse-led coaching) or short follow-ups reported mixed results [26]. Workforce capacity was a critical constraint as implementing ABM requires dedicated staff to enroll patients, manage schedules, and conduct appointments [32,33]. In rural or resource-constrained settings, pharmacists may not be available, which points to the importance of models that combine remote pharmacist consultations with local community health workers [34,35]. Reporting standardization, specifying staffing ratios, contact times, and follow-up lengths, would enable more meaningful comparisons across studies [27].

Medication synchronization and ABMs: The ABM platforms align refill dates, schedule appointments, and often include pharmacist counseling. Evidence indicated improved adherence and persistence, enhanced detection of therapy problems, and higher uptake of clinical services [22]. Synchronization reduced regimen complexity and enabled proactive outreach before gaps occurred. However, implementation requires workflow redesign and may not be feasible in settings with limited pharmacy staffing.

The heterogeneous effects based on modality and context are summarized in an evidence map (Table 1).

**Table 1:** Evidence map of targeted adherence interventions SMS: Short Message Service; LMIC: Low- and Middle-Income Country; HIC: High-income country; CVD: Cardiovascular Disease; ART: Antiretroviral Therapy; IVR: Interactive Voice Response; CHF: Chronic Heart Failure; HTN: Hypertension; PDC: Proportion of Days Covered; ABM: Appointment-Based Model (Medication Synchronization); appt: appointment; ML: Machine Learning; EHR: Electronic Health Record; BP: Blood Pressure; ROI: Return on Investment; RR: Risk Ratio; RCT: Randomized Controlled Trial; mo: months; mgmt: management; Med-sync: medication synchronization; w: weeks; pre-post: before-after design.

Modality	Targeting approach	Setting & population	Country/Region	Comparator	Outcome metric	Follow-up	Effect signal	Cost/ROI note	Key source
Two-way SMS	Gap-days; condition triggers	LMIC, mixed conditions	Sub-Saharan Africa (Kenya, South Africa)	Usual care/one-way	Adherence (PDC / self-report)	3–12 mo	Modest ↑ (RR≈1.14)	Low marginal cost	Ødegård et al., 2022 (Africa RCT meta-analysis) [14]
SMS (CVD)	Broadcast medication/lifestyle prompts	HIC, secondary prevention CVD	Australia	Usual care	Self-report adherence	6 & 12 mo	No improvement	Not primary	Chow et al, 2022 (TEXTMEDS) [15].
WhatsApp chat	Participant-tailored vs standardized	LMIC, young adults on ART	Peru (Lima)	Head-to-head designs	ART adherence (planned)	16 w	Protocol—pending	Not reported	Freidenson-Bejar et al., 2025 [36]
IVR calls	Missed-refill trigger	Primary care adults	Canada	Usual care (pilot)	Refill compliance	~3 mo	Feasible; variable	Not reported	Reidel et al., 2008 [26]
Pharmacist-led mgmt	Monthly follow-up; regimen review	CHF outpatients	China (Hebei Province)	Usual care	PDC (52 w)	52 w	Higher PDC	Not reported	Wang et al., 2024 [23]
Med-sync (ABM)	Aligned refills; appt day	Community pharmacies	Canada (Ontario)	Pre–post / control	PDC / persistence	6–12 mo	Improved adherence	Efficiency gains	Krumme et al., 2018; Waghmare et al., 2023 (systematic review) [37,38]
Mobile app + education	Risk screen; literacy-matched content	LMIC adults with HTN	Pakistan (Lahore)	Usual care	Self-report adherence; BP	12–24 w	Improved adherence/BP	Not reported	Arshed et al., 2024 [39]
ML-guided targeting	Claims/EHR features; prior gaps	Mixed health-system contexts	Multiple HIC (USA, Europe)	Rules-based vs ML	Model metrics; downstream PDC	Varied	Predictors cataloged; external validation varies	Build cost upfront	Marineci et al., 2025 (model development); Rhudy et al., 2025 (scoping review) [5,40]

Interpretation of the Evidence Base for Implementation

Strongest evidence base: Two-way SMS in low- and middle-income country (LMIC) settings (risk ratio ~1.14, low cost, supported by multiple RCTs across African countries) [13], and pharmacist-led management (consistent PDC improvements with moderate cost, demonstrated in China and other settings) [22].

Promising interventions that require further data include medication synchronization, which shows positive signals for adherence and persistence but has heterogeneous comparators [21], and WhatsApp/app-based interventions, which are still in the early stages with pending protocols [35].

Inconsistent or context-dependent: One-way SMS (null effect in cardiovascular disease (CVD) secondary prevention (Australia), positive effects in some HIV cohorts (Africa)) [14]; IVR (feasibility demonstrated in pilot studies, but effectiveness variable) [26].

Evidence gaps: Cost-effectiveness data from LMICs; head-to-head comparisons of digital vs. hybrid approaches; long-term sustainability beyond 12 months; effectiveness in middle-income countries outside of Pakistan and China [40,41].

Economics

Cost‑effectiveness analyses suggest that digital health interventions (text messaging, apps, and websites) were generally cost‑effective for diabetes and hypertension. A 2023 synthesis reported median incremental cost‑utility ratios around €3,840 per quality‑adjusted life year and recommended transparent reporting and sensitivity analyses [41]. However, cost structures and healthcare financing models vary substantially across countries; a service with favorable cost-effectiveness in one setting may not translate proportionally to another due to differences in labor costs, reimbursement systems, and infrastructure [42]. Studies of digital adherence technologies for tuberculosis indicated cost savings compared with directly observed therapy, though results varied by context [42]. Pharmacist‑led services may offer a positive return on investment, yet estimates depended on reimbursement models and are scarce for low‑resource settings. Particularly limited economic evidence from LMICs restricted generalizability.

Economic conclusions depended on the analytic perspective and horizon [43]. Direct costs included platform licensing, SMS/IVR charges, integration, and staff time to triage replies or place pharmacist calls; indirect costs included avoided travel and rehospitalizations [44]. Payers prioritized downstream utilization and adherence-linked quality bonuses; providers emphasized discharge safety and clinic throughput; and pharmacies tracked script capture and labor [45,46]. Digital outreach often showed favorable cost-effectiveness at low per-patient costs and modest adherence gains, but return on investment (ROI) for pharmacist services varied with payment model (fee-for-service vs. value-based) and case-mix [41,47]. LMIC analyses continued to be sparse; transparent reporting of unit costs for connectivity, staff, and logistics, along with scenario and probabilistic sensitivity analyses, was crucial [42,43]. Decision-makers should insist on perspective-labeled results, stated horizons, and uncertainty intervals rather than single-point ROI claims [43]. 

Equity, Privacy, and Trust

When risk models are trained on unrepresentative data, algorithmic bias can amplify disparities across race, socioeconomic status, and geography [8]. Mitigation strategies included fairness audits, feature selection that avoids proxies for protected attributes, and transparency in model performance. Data use must align with regulations; the Health Insurance Portability and Accountability Act (HIPAA) Security Rule is currently undergoing proposed updates to strengthen cybersecurity and remove distinctions between required and addressable specifications [48]. The European Union’s General Data Protection Regulation (GDPR) and Nigeria’s Data Protection Regulation (NDPR) require a lawful basis for processing, data minimization, and consent for outreach. Shared phones and limited literacy in low‑resource settings necessitate careful channel selection to protect privacy.

Equity and trust require explicit design. Subgroup reporting must be mandated for adherence outcomes and message reach; bias must be probed using PPV and FNR across key groups; and a concise fairness dashboard can be published [49]. Data can be minimized by preferring recent fills, gap days, and refill velocity over sensitive attributes; and any use of proxies can be documented [50]. Consent and opt-out should be plain-language and channel-appropriate; and shared-phone contexts call for neutral wording and IVR options [51]. The regulatory posture must be clearly noted and the HIPAA Security Rule changes must be treated as proposed until finalized; and GDPR and NDPR principles of lawful basis, transparency, minimization, and rights can be applied for access/erasure where applicable [48,52,53]. Lastly, a governance log must be maintained that records data flows, vendors, and model versions to ensure audits can verify that outreach is safe, proportionate, and compliant [49].

Discussion

Outcomes-Back Design

Effective adherence outreach begins with clear outcome targets (e.g., achieving PDC ≥80% for statins, renin-angiotensin system antagonists, or oral antidiabetics; maintaining persistence; or reducing readmissions). Minimal datasets should include recent fill dates, days of supply, gap days, comorbidities, and socio-demographics. Trigger‑based timing, such as identifying gap days or refill velocity, permits proactive messaging. Risk models with moderate performance can help allocate resources but must be evaluated for fairness [8]. Reporting persistence alongside PDC and specifying denominators (e.g., class switches) improves comparability [4].

Channel Strategy by Resource Context

In low-resource settings, SMS, USSD, and IVR remain the backbone of outreach. Utilizing these channels alongside clinic-linked pharmacy prompts or community health worker visits can help overcome literacy barriers and issues related to device sharing. Evidence from two‑way messaging trials showed that interactive communication yielded modest improvements [14]; however, null results in some cardiovascular cohorts remind us that content and timing matter [7]. High-resource environments can integrate smartphone apps, patient portals, and synchronized refills, enabling automated risk stratification and real-time adherence monitoring. Yet privacy expectations may differ: smartphone users might tolerate app notifications, whereas shared phones require discreet voice calls or community‑based visits. Decision trees considering connectivity (smartphone vs. feature phone vs. landline), privacy sensitivity, and literacy can help select appropriate channel bundles.

Human‑in‑the‑Loop: Pharmacists, Care Coordinators, and Appointment‑Based Models

Pharmacists play a pivotal role in closing adherence gaps. Their ability to reconcile regimens, adjust therapy, provide education, and coordinate care explains why pharmacist‑led interventions often improve adherence [23]. The ABM schedules all medication refills for a single date and includes a pharmacist appointment, which leads to improved PDC, increased persistence, and the identification of therapy problems [22]. However, the impact varied by setting and patient population. For instance, the 2025 electronic outreach study showed improvement only for diabetes medications [2]. Integration with care coordinators and primary care teams can ensure that nonadherent patients receive timely human contact when automated messages flag concerns.

Design for Behavior: Message Content, Timing and A/B Learning

Behavior-change techniques such as goal setting, framing messages from credible sources, providing feedback, and prompting commitment can enhance adherence. Messages should be timed close to expected refill dates and incorporate two‑way loops for patients to ask questions or report barriers. A/B testing of message wording, timing, and channel can identify effective combinations; preregistration of outcomes and transparency about neutral results prevent publication bias. Adaptive algorithms can optimize message libraries over time while ensuring fairness and avoiding reinforcement of disparities [14].

Fair, Safe, and Compliant Targeting

Model developers should conduct fairness audits to detect and mitigate bias [8]. Consent processes must be transparent about data usage, the purpose of outreach, and opt-out mechanisms. The proposed HIPAA Security Rule would require more specific cybersecurity safeguards and the elimination of the “addressable” category [48]. Under the GDPR and NDPR, having a lawful basis, ensuring data minimization, and conducting privacy impact assessments are mandatory requirements. Outreach programs should also take into account the cultural norms prevalent in communities where people share phones. Messages regarding sensitive conditions may necessitate the use of privacy-preserving channels. Regular monitoring of model performance across subgroups and publication of fairness dashboards promote accountability.

Economics and Sustainability

Resource allocation is critical for sustained adherence programs. Digital outreach through SMS or apps incurs low marginal costs and is generally cost‑effective [41]. Pharmacist‑led services may generate savings by preventing complications but require reimbursement mechanisms; cost‑benefit analyses show mixed results, often depending on payer perspective and integration with value‑based payment models. ABMs can improve efficiency by bundling refills and reducing travel expenses. Gaps remain in the economic literature from low‑ and middle‑income countries and for interventions that combine human and digital components. Future studies should report detailed cost components, perspectives (payer, provider, or societal), time horizons, and include sensitivity analyses.

Implications for Clinical Leadership, Pharmacy Services, and Payers

Healthcare leaders should adopt a risk‑tiered playbook. Tier 1 triggers on gap days and sends low‑intensity SMS or IVR messages, reserving pharmacist time for high‑risk patients. Tier 2 combines proactive two-way messaging with ABM: pharmacists review regimens, adjust therapy, and coordinate with prescribers. Tier 3 integrates care coordinators for patients with complex comorbidities, addressing social determinants and deprescribing. Clinics must enroll high‑risk cohorts, build message libraries grounded in behavioral science, and set measurement cadences. Community pharmacies should embed ABM and integrate targeted prompts at refill events [22]. Payers, particularly Medicare Advantage plans where adherence metrics carry triple weightings, need to align benefits with PDC star measures and audit models for equity; and targeted outreach can close gap days while safeguarding fairness [2].

The playbook must be implemented in a stepwise manner. Begin by identifying priority drug classes and cohorts with the highest clinical and financial impact; assemble a minimal dataset (fill history, days’ supply, gap days, switches); and select a channel bundle matched to connectivity, privacy, and literacy. Pre-register key performance indicators (KPIs; PDC threshold attainment for target classes and persistence) and equity cuts [3,54]. Launch with Tier 1 triggers (gap days + two-way SMS/IVR), escalate to Tier 2 (pharmacist medication synchronization and counseling) for persistent gaps, and reserve Tier 3 (coordinated medication review/deprescribing) for complex regimens [14,22,23]. Review dashboards monthly for signal and quarterly for calibration and fairness; retire weak variants, scale those with durable gains, and document changes in a change-control log [49].

Future Recommendations

Randomized trials should pair risk‑stratification with the human touch, comparing digital and hybrid interventions across diverse settings. Studies must report PDC, persistence, and equity outcomes by subgroup and preregister A/B testing protocols. Researchers should publish message libraries and share anonymized data to facilitate replication. Model governance checklists and fairness dashboards can aid compliance with emerging regulations [48]. Cross-jurisdictional privacy playbooks should interpret HIPAA, GDPR, and NDPR requirements for targeted outreach. Economic evaluations, particularly in LMICs, should include quality‑adjusted life years or disability‑adjusted life years and perform full sensitivity analyses [41].

Limitations

This review employed a narrative design and thus could not provide pooled effect estimates or causal inferences. Studies varied widely in their definitions of adherence and persistence, measurement intervals, and follow-up lengths, complicating comparisons. PDC and MPR may overestimate adherence when stockpiling or auto‑refills occur and may underestimate persistence when patients switch agents within a class. Moreover, only English‑language publications were included; hence, data from non‑English sources and unpublished program evaluations were missing. Rapid advances in digital health and evolving regulatory landscapes mean that evidence may quickly become outdated.

## Conclusions

Targeted, data‑driven patient engagement can improve medication adherence when thoughtfully designed and implemented. Risk‑stratification models using claims or behavioral data help allocate resources but must be externally validated and audited for fairness. Two‑way messaging and pharmacist integration yield the most consistent improvements, whereas one‑way reminders alone often have limited impact. ABMs that synchronize refills and embed pharmacist appointments show promise across settings. Cost‑effectiveness generally favors digital outreach, yet economic data from low‑resource contexts remain sparse. Future programs should combine predictive analytics, behaviorally-informed content, human support, and robust governance to enhance adherence while protecting equity and privacy.
